# Quinine

**Published:** 1889-11-09

**Authors:** 


### THERAPEUTICS.

### QUININE.

The value of large doses (gr.5 and upwards) of quinine, not only in malarial diseases, comparatively rare in this country, but not less in neuralgia, especially facial, is so great that any means of averting the distressing symptoms of cinchonism, which four or five grains will induce in many persons, would be hailed by patients and practitioners. Italian physicians have an experience of quinine second only to that of our Indian brethren, and Dr. Coglitore states that he has succeeded in the gravest cases of paludal fever by combining it with ergotin and a small proportion of opium. His formula is ℞. quiniæ sulph. 75 centigrams, Bonjean's ergotin 30 centigr., opii. 5 centigr. Make three powders, to be taken at intervals of an hour. The English equivalents are: quin. grains 12, ergotin gr. 5, opium gr. ⅘. He has no faith in the so-called substitutes for quinine, arsenic, salicin, eucalyptus, etc. Coglitore's combination is well worth trying in that very common ailment, facial neuralgia from an unsound tooth, in which, where there is no periostitis, we have found two or three five-grain doses of quinine taken in the course of a couple of hours almost always successful.

